# Behavioural Distinction between Strategic Control and Spatial Realignment during Visuomotor Adaptation in a Viewing Window Task

**DOI:** 10.1371/journal.pone.0048759

**Published:** 2012-11-16

**Authors:** Jane M. Lawrence-Dewar, Lee A. Baugh, Jonathan J. Marotta

**Affiliations:** Perception and Action Laboratory, Department of Psychology, University of Manitoba, Winnipeg, Manitoba, Canada; University of California, Davis, United States of America

## Abstract

We must frequently adapt our movements in order to successfully perform motor tasks. These visuomotor adaptations can occur with or without our awareness and so, have generally been described by two mechanisms: strategic control and spatial realignment. Strategic control is a conscious modification used when discordance between an intended and actual movement is observed. Spatial realignment is an unconscious recalibration in response to subtle differences between an intended and efferent movement. Traditional methods of investigating visuomotor adaptation often involve simplistic, repetitive motor goals and so may be vulnerable to subject boredom or expectation. Our laboratory has recently developed a novel, engaging computer-based task, the *Viewing Window,* to investigate visuomotor adaptation to large, apparent distortions. Here, we contrast behavioural measures of visuomotor adaptation during the *Viewing Window* task when either gradual progressive rotations or large, sudden rotations are introduced in order to demonstrate that this paradigm can be utilized to investigate both strategic control and spatial realignment. The gradual rotation group demonstrated significantly faster mean velocities and spent significantly less time off the object compared to the sudden rotation group. These differences demonstrate adaptation to the distortion using spatial realignment. Scan paths revealed greater after-effects in the gradual rotation group reflected by greater time spent scanning areas off of the object. These results demonstrate the ability to investigate both strategic control and spatial realignment. Thus, the *Viewing Window* provides a powerful engaging tool for investigating the neural basis of visuomotor adaptation and impairment following injury and disease.

## Introduction

We must frequently adapt our movements to successfully complete everyday tasks. These adaptations are sometimes conscious modifications in our motor plan in reaction to large distortions in order to achieve a desired output. At other times, the altered movements may be so subtle that we may not be aware they are occurring. Two mechanisms of visuomotor adaptation have generally been agreed upon: strategic control and spatial realignment. These mechanisms have also recently been described as ‘fast’ and ‘slow’ processes of motor adaptation [1]. Strategic control, or the fast component, involves a recalibration or the selection and learning of specific movements in order to successfully complete the desired action [2]. Whereas spatial realignment, or the slow component, is an automatic spatial remapping due to a detected discrepancy between an intended movement and the resulting sensory feedback [3], [4], [5]. During tasks of subtle distortions, it is believed that proprioceptive sensory feedback updates an internal model [6]. As an example of these two strategies, a car may develop a subtle and progressive problem with the steering column. A person who drives the car everyday may not notice this issue because as the distortion progresses over time, they automatically adapt their movements to match – this is spatial realignment. In contrast, a person who seldom uses that car may notice the distortion right away and adjust the direction of the steering wheel in order to compensate for this change – this is strategic control.

Visuomotor adaption has classically been studied through prism experiments [7]. In these studies, subjects view objects through prism lenses, which alter the perception of an object’s location. Healthy individuals can adapt their motor movements to accommodate the dissociation between their visual perception and the object’s actual location. More recently, computer based tasks have supplemented these experiments and allowed more flexibility in their design as well as the acquisition of more precise and detailed behavioural information. In general, these tasks have tended to use target-based pointing [8] movements.

By introducing rotational distortions either incrementally, or suddenly, behaviour during spatial realignment and strategic control has been investigated during prism studies [9] and computer based point-to point or target tasks [8], [10], [11]. Kagerer et al [8] used a digitized point-to-point task on a touch sensitive tablet to show that participants exposed to a gradual, incremental rotation of 10°, up to a maximum of 90°, demonstrated faster movements with less spatial error and showed larger after effects than those who experienced a 90° rotation throughout the experiment. In their task, participants moved a marker to one of four targets while having no vision of their moving arm or hand. Klassen et al. [10] used an out-and back target task to one of eight targets where in a gradual condition increments of 0.125° were introduced to a maximum of 30° in a gradual distortion group. Even though these tasks have revealed important information regarding visuomotor adaptation behaviour and its neural basis, they are simplistic as they require one trajectory of movement therefore limiting the internal model a participant is capable of building during adaptation. In order to investigate visuomotor adaptation in a more natural, enriched setting, our lab has developed a computer-based task, the *Viewing Window* [12], [13], [14]. The paradigm is a visuomotor experiment hidden within a perceptual task. Subjects view a blurred or masked image of an object on a computer screen with the instruction to identify the object as quickly and accurately as possible. To accomplish this, they must move a small, circular region, the *Viewing Window,* around the screen using a touch sensitive screen, trackball or joystick. The task has previously been used during reading and text comprehension tasks [15], [16], as well as scene exploration [17], to investigate visual attention and perception. More recently, our lab has used to this task to investigate visuomotor adaptation by manipulating the relationship between the actions of the participant and the resulting movement of the window [14]. By restricting the region of an object that can be viewed clearly, the visuomotor scanning pattern can be analyzed. The benefits of the *Viewing Window* over traditional prism experiments is that this task requires minimal experimenter instruction, distortions can be presented in finer and progressive increments, it can be performed on a compact tablet PC therefore it is highly portable, and precise timing and spatial information regarding viewing window movement and object identification can be recorded.

Our previous studies with the *Viewing Window* task have utilized large distortions in the form of flips in *Viewing Window* movement in the horizontal and vertical direction and so it would be anticipated that strategic control would be the predominant mechanism of visuomotor adaptation [12], [13], [14]. The use of this task has revealed novel recruitment of the claustrum, not found in previous studies of visuomotor adaptation [12]. The purpose of the present study is to demonstrate that the *viewing window* paradigm can be used to investigate both strategic control and spatial realignment.

We hypothesize that distinct behaviours between participants using strategic control and those who adapt using spatial realignment can be measured using the *Viewing Window* paradigm. In line with our previous Viewing Window studies, we anticipate participants in the sudden rotation group will demonstrate difficulty in controlling the window when the distortion is initially introduced, which will be reflected by more complicated paths of movement, slower velocities of movement, and more time spent off the object during the first half (early phase) of the trials. As the experiment progresses, we hypothesize the participants will adapt and their performance evaluated by these measures will improve during the second half of the distortion trials (late phase) and will continue to do so once the distortion is removed. In contrast, we hypothesize that participants in the gradual rotation group will not notice the introduced distortion and so changes in behaviour in the form of movement velocities, the time taken to identify the viewed object (scan time), and the time that the *Viewing Window* spent on areas that did not contain object image (time off object), will be minimal during the early and late phase distortion trials and the paths of window movement will be more simple with fewer changes in direction compared to the sudden rotation group. However, once the distortion is removed in the post-distortion phase, we hypothesize that participants in the gradual rotation group will show greater after-effects in the form of decreased performance of these measures. The ability to distinguish and measure behavioural differences between groups will demonstrate that the Viewing Window task can be used to evaluate performance of both strategic control and spatial realignment.

The ability to use the *Viewing Window* to measure behaviour during strategic control and spatial realignment would provide a novel, powerful tool for examining visuomotor adaptation. This would expand the repertoire of experimental designs available for investigating visuomotor adaptation in healthy individuals and in decline due to natural aging, and neural injury and degenerative disease.

## Methods

### Subjects

Written informed consent was obtained from all subjects prior to the study. Fifty-eight right-handed, first year psychology students with normal or corrected-to-normal vision (15 male) were recruited from the University of Manitoba psychology participant pool. Subjects received course credit for participating in the study. This research was approved by the Human Research Ethics Board at the University of Manitoba.

Prior to start of the experiment, subjects were familiarized with the response equipment, a track ball, used in the study. Participants were presented with a medium difficulty maze (set 5) acquired from www.printablemazes.net and asked to guide a computer cursor through the maze to get acquainted with the sensitivity of the trackball. Participants were aware that no data were being collected during this time and that it was self-paced.

### The Viewing Window Task

**Figure 1:**
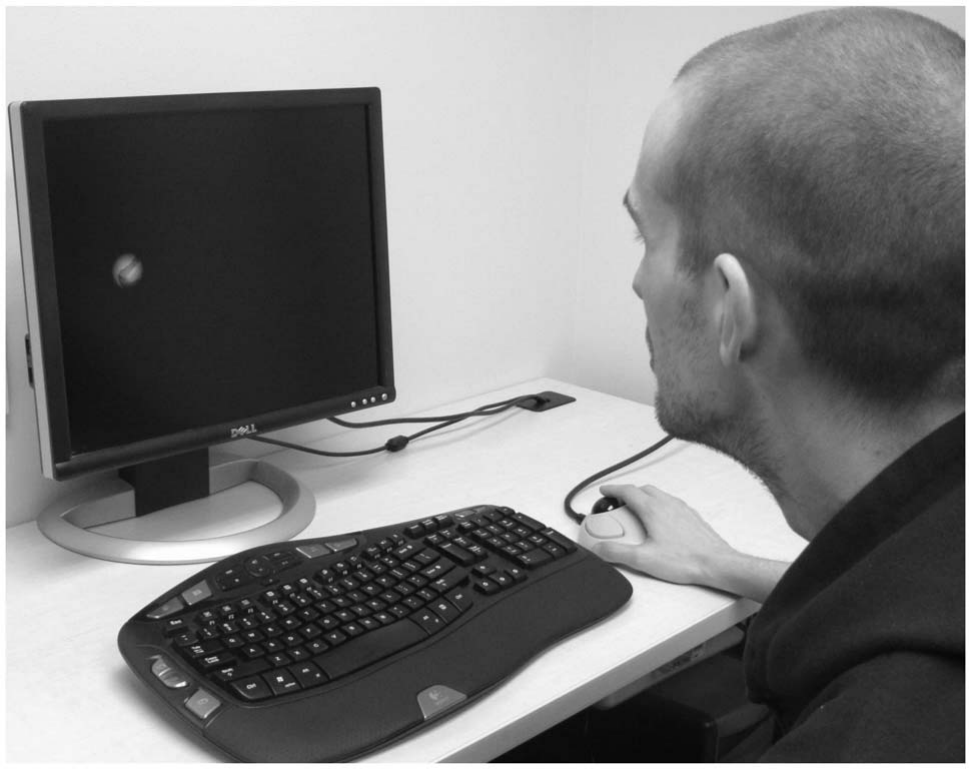
Experimental setup of the *Viewing Window* task. Subjects sat at a desktop workstation and viewed masked images on a 19″ monitor. In order to identify the images, subjects used a trackball to control the movement of the Viewing Window that would allow part of the image to be seen clearly. Subjects were instructed to, upon identification of the image, press the space bar on the keyboard and enter the name of the object in a text box that would appear on the computer screen. The photographed subject has given written informed consent, as outlined in the PLoS consent form, to publication of their photograph.

**Figure 2:**
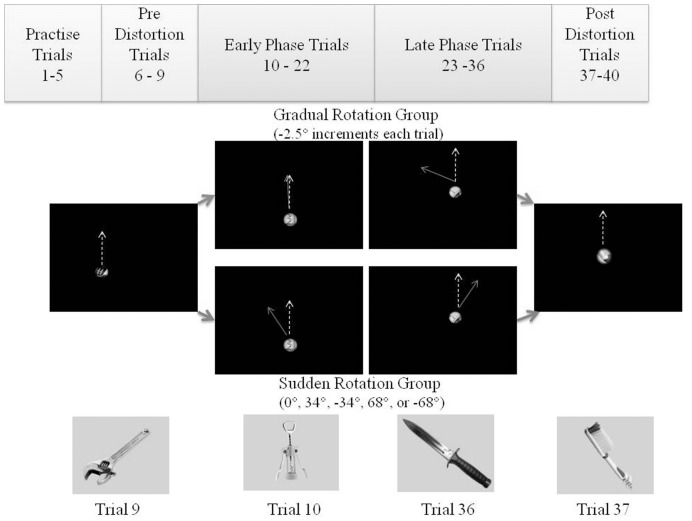
Experimental design of the *Viewing Window* task. The first five trials were used as practise and therefore excluded from all analysis. No distortion was present in movement of the *Viewing Window* during trials 6–9. During trials 10–36, in two separate groups, participants either received a gradual progressive distortion of 2.5° in the counter-clockwise direction on each trial, or a large sudden distortion in either direction. For analysis, these trials were divided in half in two phases (early and late). On trial 37 the distortion was removed and control of the *Viewing* Window returned to normal for the remainder of the study to examine after-effects.

The *Viewing Window* task [14], is an in-house software program written in Matlab® (2007b, MathWorks, Natick, MA), run on a Dell tablet computer and presented to the participant on a 19″ LCD monitor. During the present study our methods were modified slightly from our previously published studies [12], [13], [14]. During pilot testing, the sudden rotation distortions were not apparent to all subjects when a blurred image was presented. Therefore, clear images of the objects were overlaid with a black mask. As a result, subjects could not obtain any additional cues as to where useful information may be, resulting in a much more difficult task. The number of trials therefore had to be reduced to maintain an acceptable duration of experiment and prevent subject fatigue. In the present study, 40 trials were presented which all subjects completed in less than one hour. During each trial a masked picture of an everyday object would appear at 1280 × 1024 resolution. All participants viewed the same 40 images however, to control for order effects, 2 counterbalanced lists were used so that half the participants in each experimental group would view the list in one order, and the other half would view the list in the reverse order. No images were viewed more than once by each participant. Even though there was some variation in the size of the image of the object, all images were larger than the size of the Viewing Window so that no object could be seen in its entirety without moving the Window. The goal of the task was to identify the object as quickly and accurately as possible. To do this, subjects used a trackball to control the *Viewing Window,* a small circular region through which part of the object could be seen completely clearly (Figure 1). During the first 9 and last 4 trials the movement of the viewing window was controlled normally by the trackball (Figure 2). During the 27 trials in between, a distortion was introduced between the movement of the trackball and the resulting movement of the *Viewing Window*. Subjects were divided into two groups according to the way in which this distortion was presented. To evoke strategic control, distortions had to be presented large enough to be noticeable, and in enough variety to avoid becoming predictable. In a previous pilot study we had utilized two large distortions in the counter-clockwise direction, pseudo-randomized with no distortion. However, to an adapted subject, a change from a counter-clockwise distorted trial to one with no distortion is similar to adapting to a change in the clockwise rotation. More problematic was that with only three conditions it was difficult to pseudo-randomize the change in control from one trial to another. In the present study, we aimed to pseudo-randomize the change experienced by the subject and so used two large distortions, but in either direction (four in total). Therefore, the group in which we aimed to evoke strategic control in, received large noticeable distortions of 0°, 34°, or 68° in either direction, so that the change in rotation from the previous trial was pseudo-randomized. In the second group, distortions incrementally increased in each trial by 2.5° in the counter-clockwise direction. In this distortion group, changes were cumulative in one direction as we wanted to end the distortion period at a large rotation, so that return to normal control in the post-distorted trial would be evident.

Subjects received verbal instruction to try to identify the images as quickly and accurately as possible by using the trackball to move the window around the computer screen. Once subjects knew the identity of the object, they were to press the space bar on a keyboard and enter the name in a text box that appeared on the computer screen. The task would then move onto the next trial with a new masked image. The task therefore proceeded at the subject’s own pace. Feedback regarding accuracy was not provided to the participant.

To account for order effects, two lists of objects were used that were counterbalanced. Therefore, in each rotation group, ten subjects received one list of items and the other ten received the same list but the order of items in the experimental (distortion) period was reversed.

### Data Analysis

All participants knew that the task was self-paced and so while they received the instruction to identify the object “as quickly and accurately as possible”, time was not a limiting factor. It is possible that participants, motivated by the goal to identify the object, guessed at the object identity. To account for this, an accuracy threshold of 60% was applied to consider data from participants who were fully engaged in the task and the highest performing. This type of threshold was selected because it was the most objective. The use of a threshold more specific to the motor component of the task rather than the perceptual component may be more desirable in examining adaptation behaviour such as movement velocity, or the time off the object. However, establishing the level of this threshold is subjective. This is an interesting topic for further examination. As a result of the accuracy threshold, 18 subjects were excluded and so a total of 58 participants had to be recruited to obtain group sizes of 20 subjects for each experimental group. The resulting data analyzed was from 40 participants (14 male, mean age 19.5±1.9 years, range 18–26 years). An additional analysis of all 58 participants is presented in Supplementary Information S1.

The first five trials were removed as practice trials. The remaining data sets were exported to SPSS 13.0. Data was filtered to exclude trials in which the object was incorrectly identified and to consider only data acquired after movement of the window was first initiated for each trial. Measures of movement behaviour examined included the velocity of the Viewing Window, time the image was viewed (scan time), and the percentage of the time the Viewing Window spent on the object. For each subject, trials were identified as outliers by calculating the Z scores for each of the examined behavioural measures and excluding data beyond 2.5 standard deviations from all subsequent analyses. To examine differences between groups during the distortion trials, the movement velocities, scan times and percentage of time spent off the object were analyzed with ANOVA with Bonferroni correction (alpha 0.05). To examine changes in behaviour over the course of the experiment the trials were then divided into the pre-distortion phase (trials 6–9), early distortion phase (trials 10–22), late distortion phase (trials 23–36), and post distortion phase (trials 37–40). To examine phase interactions within groups, a repeated-measures ANOVA was conducted. For each subject, data were normalized by subtracting the mean of the appropriate behavioural measure obtained during the pre-distortion period. For example, to normalize the measurement of mean scan time during the early distortion period for subject X, the mean scan time found during the pre-distortion phase (trials 6–9) was subtracted from the mean scan time found during the early distortion phase (trials 10–22). This process was repeated for each phase, for each behavioural measure, and for each subject. The normalized data were then analyzed with a repeated measures ANOVA with Bonferroni correction using a factor structure for each behavioural measure of a 2 [Group:Gradual vs Sudden distortion] × 3 [Phase: Early, Late, Post distortion]. The scan paths of *Viewing Window* movement were visually inspected.

## Results

**Figure 3:**
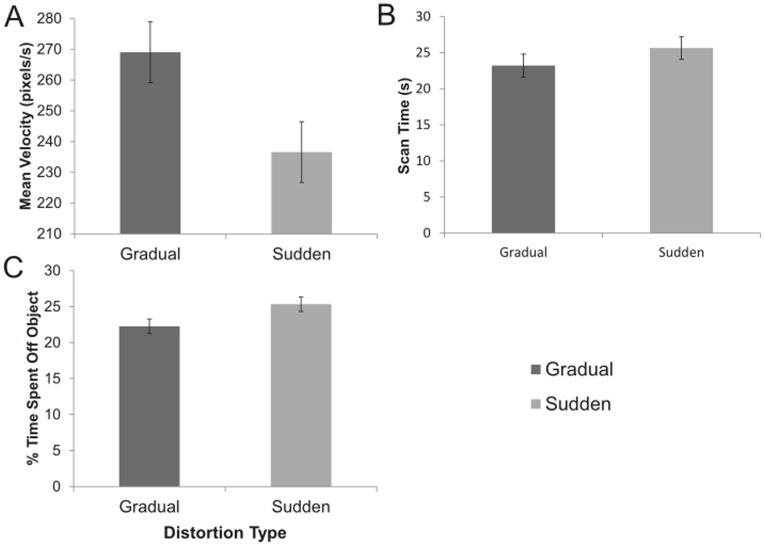
Comparisons of behavioural measures between groups during distorted trials. A) Subjects in the gradual rotation group demonstrated significantly faster (F(1,38) = 5.406, p<0.05) mean velocities than those in the sudden rotation group. B) Subjects in the sudden rotation group spent a slightly longer (but not significant) time scanning the images prior to indicating their identification. C) Subjects in the sudden rotation group spent significantly more time (F(1,38) = 4.620), p<0.05) with the *Viewing Window* off of the object than those in the gradual rotation group.

Group comparisons revealed several anticipated behavioural differences (Figure 3). Subjects in the gradual rotation group demonstrated significantly faster mean velocities of movement compared to the sudden rotation group (F(1,38) = 5.406, p<0.05 Figure 3A). No significant difference in scan times were identified (F(1,38) = 1.185, p>0.05, Figure 3B). The gradual rotation group spent significantly less time scanning areas off the object (F(1, 38) = 4.620, p<0.05, Figure 3C). The faster velocities and ability to keep the window on the object, suggest that the gradual rotation group experienced less difficulty in controlling the movement of the window and adapted easily to the distortions. The slower movement velocities and greater time off the object observed in the sudden rotation group suggest that participants did experience some difficulty in controlling the *Viewing Window* movement.

**Figure 4:**
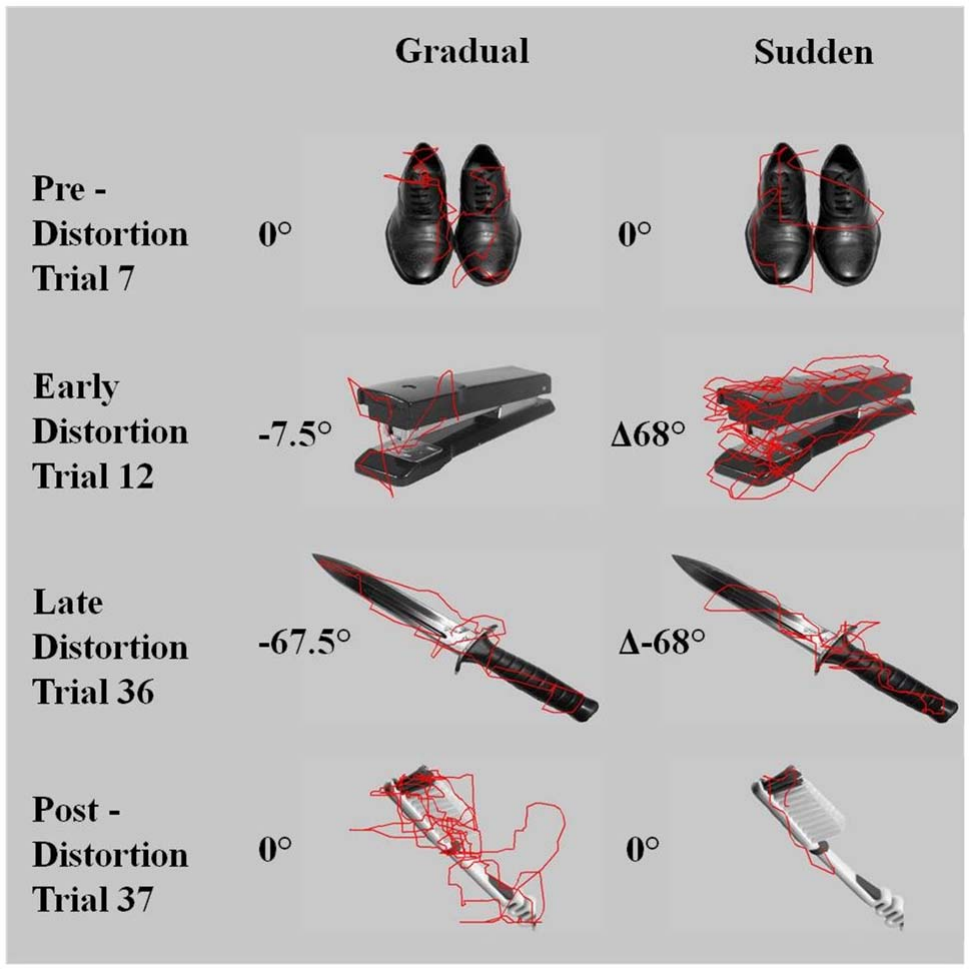
Sample scan paths from representative participants. A) gradual rotation group and B) sudden rotation group. The line represents the pathway of the viewing window from image onset until participant signal of image identification.

When data were divided into pre, early, late, and post distortion periods, several behavioural differences between groups were visible. Figure 4 depicts sample scan paths from one representative subject in the gradual rotation group and another representative subject from the sudden rotation group. The gradual rotation subject demonstrates fairly simple scan paths during the distortion trials indicating good control over window movement. However, once the distortion is removed, the same participant shows after-effects in the form of difficultly in keeping the *Viewing Window* on the object. In contrast to this behaviour, the participant in the distortion group shows a complicated scan path and therefore more difficulty when the distortion is first introduced. By the last distortion trial, the same participant has adapted and shows a simpler scan path with fewer changes in direction. When the distortion is removed, the sudden rotation subject shows a much smaller after-effect compared to that observed in the gradual rotation subject.

**Figure 5:**
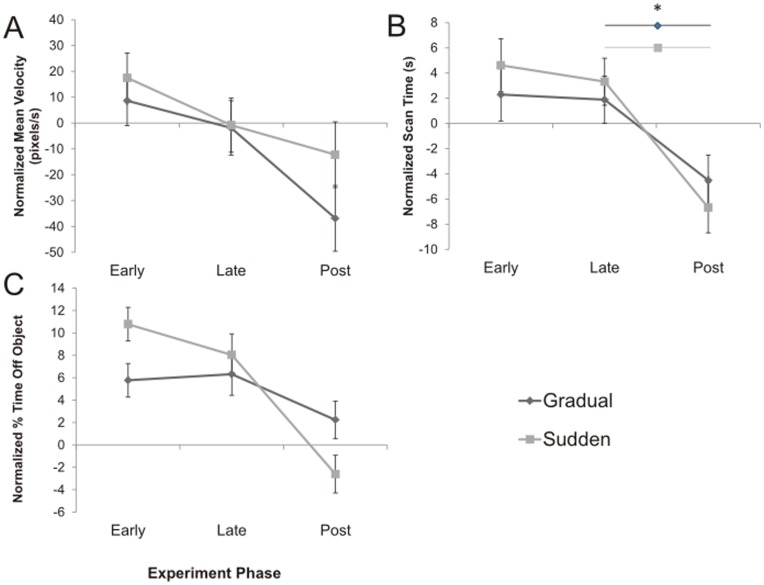
Normalized mean behavioural measures by phase. Behavioural measures of the early, late, and post distortion values are presented relative to the baseline (measures during the pre distortion phase) of each group. A) Velocity of Viewing Window movement. B) Scan Time required to identify object *Both distortion groups demonstrated a significant reduction in scan time in the post distortion phase (p<0.05). C) Percentage of Time off Object. A significant interaction was found between phase and distortion type was found in the percentage of time spent off the object (p<0.001).

Quantitatively, these observations can also be made with the examined behavioural measures. Both groups demonstrated reductions in movement velocity that were significant by phase (Figure 5A, F (2,76) = 14.890, p<0.01). Overall, there was not a significant interaction between phase with distortion type for mean movement velocity (F (2,76) = 1.486, p>0.05). Relative to the pre distortion period, a slight (but not significant, p = 0.078) increase in scan time was observed in the sudden rotation group in the early phase (Figure 5B). Both groups demonstrated significant reductions in scan times in the post distortion phase (p<0.05), however, this reduction was slightly larger in the sudden rotation group. Overall, there was a significant effect of phase (F (2,76) = 43.396 = p<0.01) but not a significant interaction between phase with distortion type for mean scan time (F(2,76) = 2.463, p>0.05). A significant interaction between phase and distortion type was found for the amount of time that the *Viewing Window* spent off the object (F(2,76) = 10.320, p<0.001, Figure 5C). Relative to the early phase, subjects in the sudden rotation group demonstrated greater difficulty in controlling *Viewing Window* movement compared to those in the gradual rotation group as evidenced by a greater percentage of time spent off the object (Figure 5C). Both groups demonstrated significant differences compared to their baseline values (p<0.05). During the post distortion phase, subjects in the sudden rotation group spent significantly less time off the object (p<0.05).

## Discussion

We hypothesized that the *Viewing* Window task could be used to investigate two mechanisms of adaptation. To do this, we identified measurable differences in behaviour in two experimental groups eliciting strategic control and spatial realignment. When comparing behavioural measures during all distortion trials of the two groups, we found significant differences in the movement velocities, and time that the *Viewing Window* spent on areas in which there was no object. This demonstrates the ability to distinguish quantitative differences in behaviour between the use of strategic control and spatial realignment within a novel, engaging computer-based task for studying visuomotor adaptation. *No significant difference was observed between scan times however, this aspect of behaviour is also influenced by the perceptual component of the task. While the existence of between group differences during distortion trials is interesting, of more relevance is how behaviour changes over the course of study, therefore, an examination of behaviour by phase was also conducted.*

When the trials were divided into four phases: pre, early, late, post distortion phases; no significant interaction between distortion type and phase of the experiment were found for movement velocities and scan times. However, a significant effect of phase was found. We anticipate that scan time is not only influenced by the ability to move the *Viewing* Window to the desired areas of the computer screen but also by the participant’s familiarity with the type of object. The item list contained a variety of everyday objects that most people would encounter including, fruits, vegetables, tools, office supplies, and musical instruments. However, how quickly these are identified can be expected to be influenced by how often a participant encounters these particular items. For example, a person who does not often interact with tools may have more difficulty identifying the image of the vice grips. The ability to recognize the objects may also be influenced by the perspective of the images. The items were displayed with variable orientation which could affect the difficulty of the task. Even though this strengthens the task in terms of increasing the variability of the location of valuable information on the computer screen, the changing perspective could also affective the ability to identify the objects. This is not something that was controlled, however, it was one reason for using counterbalanced lists in the two groups so that any effects would not drive the results of any particular phase of the study. In addition, it is possible that participants may have improved their scan times over the course of the experiment as they because more familiar with the task. We excluded the first items to account for practice trials. It is possible that subjects are more likely to hesitate in signalling their response at the start of the experiment while the task is still novel and answer more quickly later in the study when they become more confident. These influences contributed to the observation of decreased mean scan time between the late and post distortion phases (Figure 5B) despite the mean velocities also decreasing (Figure 5 A). The decreased velocity indicates increased difficulty in controlling the window movement, however, participants were still able to quickly identify the masked objects. In the analysis of time spent off the object, a significant difference between groups was found indicating larger after effects in the gradual rotation group.

The present study demonstrates that the *Viewing Window* is capable of distinguishing performance of both strategic control and spatial realignment and therefore is a powerful tool for measuring visuomotor adaptation behaviour. Even though subjects in the gradual rotation group did not report observing a change during the distortion trials, the large after-effects observed in their scan behaviour indicate adaptation took place. This is consistent with previous studies investigating gradual rotations [8].

In addition to behavioural differences, the neural basis of strategic control and spatial realignment are believed to differ. Strategic control primarily recruits posterior parietal cortex [18], [19], whereas, spatial realignment is believed to rely on the cerebellum [4], [20], [21], [22]. However, to date there have only been a handful of neuroimaging studies investigating the neural correlates of visual motor adaptation [18], [23], [24], [25], [26]. We have recently employed the *Viewing* Window task with large distortions in the form of flips in direction to investigate the neural basis of strategic control using functional magnetic resonance imaging (fMRI) and found in addition to traditional areas associated with visuomotor adaptation, novel recruitment of the claustrum. This task presents several advantages over other tasks previously used as it is computer-based therefore easier to manipulate a range of distortions over a short period of time. This would be difficult to do using prism glasses in an environment such as that during a functional magnetic resonance imaging (fMRI) study. In the future, we will utilize this paradigm to investigate the neural correlates that underlie both mechanisms of visuomotor adaptation.

Incremental rotations have previously been used to investigate visuomotor adaptation without the awareness of the participant [10], [11], [25]. However, there are some differences between our experimental design and those used in the past. Kagerer et al. [8] used a rotated mouse task with 10° increments during their gradual rotation condition to a maximum rotation of 90°. In their study, they suggest that using a gradual feedback distortion allows for a more complete adaptation compared to a sudden onset of the distortion [8]. During our 27 trials of distortion we kept increments as small and uniform as possible in order to ensure that distortions would not be apparent to the subject. Therefore, we selected an incremental change of 2.5° to a maximum rotation of 67.5°. The present study task differs from both Kagerer et al [8] and Klassen et al [10] in that subject naturally explored the computer screen without restrictions on directions. Kagerer et al [8] used a target task where subjects used a pen to start a center point and were instructed to draw a line in one of four directions. Similarly, Klassen et al [10] used a point-to-point task in eight directions. The use of the *Viewing Window* task has several advantages over previous prism studies and anti-pointing tasks. First, the adaptation task is hidden within a perceptual task. Subjects do not know that the motor relationship is altered ahead of time. Second, the *Viewing Window* investigates visuomotor adaptation in a more natural visual scanning environment in which the directions of motion are not restricted. In point-to-point tasks, subjects make directed movements in a restricted number of directions. One may anticipate that the increased freedom in direction used in the present study allows for more detailed remapping and would contribute to more complete adaptation.

The results of the present study broaden the research applications of the *Viewing Window* as a tool for investigating disturbances in spatial realignment more specifically. This is not only useful in the context of changes due to natural aging but also in the study of impairment following injury and neurodegenerative disease. The use of a visuomotor rotation task has previously been used in patients with cerebellar degeneration [27]. Computer-based point-to-point tasks have previously been used for investigating adaptation behaviour in children with Developmental Coordination Disorder (DCD) [28]. Historically, rehabilitation methods using prism lenses have shown to be beneficial for patients with unilateral neglect (see [29] for review). The development of novel, valid techniques that are simple to administer may advance rehabilitative medicine for these conditions. A restricted focus task such as the Viewing Window may also show to be beneficial in the assessment and rehabilitation of patients with unilateral neglect or hemianopia. The application of the *Viewing Window* within patient work may help further elucidate the roles of specific cerebellar structures in visuomotor adaptation in a more natural setting. We are currently exploring the use of the *Viewing Window* as part of a dual-task paradigm in a combined cognitive/motor rehabilitation therapy program. In summary, we demonstrate that distinct behaviours used during strategic control and spatial realignment can be measured using the *Viewing* Window. This provides a powerful, engaging tool for investigating the neural basis of visuomotor adaptation and impairment following injury and disease.

## Supporting Information

Supporting Information S1 **Supplementary methods and results.** We examined behaviour of all of the 58 recruited participants in a separate analysis. The analysis methods and results are described.
(DOCX)

## References

[1] Keisler A, Shadmehr R (2010) A shared resource between declarative memory and motor memory. The Journal of neuroscience 30: 14817–14823. 21048140 10.1523/JNEUROSCI.4160-10.2010 PMC3379001

[2] Redding GM, Rossetti Y, Wallace B (2005) Applications of prism adaptation: a tutorial in theory and method. Neuroscience and biobehavioral reviews 29: 431–444. 15820548 10.1016/j.neubiorev.2004.12.004

[3] Redding GM, Wallace B (1996) Adaptive spatial alignment and strategic perceptual-motor control. Journal of experimental psychologyHuman perception and performance 22: 379–394. 10.1037//0096-1523.22.2.379 8934851

[4] Weiner MJ, Hallett M, Funkenstein HH (1983) Adaptation to lateral displacement of vision in patients with lesions of the central nervous system. Neurology 33: 766–772. 6682520 10.1212/wnl.33.6.766

[5] Redding GM, Wallace B (2006) Generalization of prism adaptation. Journal of experimental psychologyHuman perception and performance 32: 1006–1022. 10.1037/0096-1523.32.4.1006 16846294

[6] Roby-Brami A, Burnod Y (1995) Learning a new visuomotor transformation: error correction and generalization. Brain researchCognitive brain research 2: 229–242. 8580736 10.1016/0926-6410(95)90014-4

[7] Rock IG, Goldberg J, Mack A (1966) Immediate correction and adaptation based on viewing a prismatically displaced scene. Perception and Psychophysics 1: 351–354.

[8] Kagerer FA, Contreras-Vidal JL, Stelmach GE (1997) Adaptation to gradual as compared with sudden visuo-motor distortions. Experimental brain researchExperimentelle HirnforschungExperimentation cerebrale 115: 557–561. 10.1007/pl00005727 9262212

[9] Michel C, Pisella L, Prablanc C, Rode G, Rossetti Y (2007) Enhancing visuomotor adaptation by reducing error signals: single-step (aware) versus multiple-step (unaware) exposure to wedge prisms. J Cogn Neurosci 19: 341–350. 17280521 10.1162/jocn.2007.19.2.341

[10] Klassen J, Tong C, Flanagan JR (2005) Learning and recall of incremental kinematic and dynamic sensorimotor transformations. Experimental brain researchExperimentelle HirnforschungExperimentation cerebrale 164: 250–259. 10.1007/s00221-005-2247-4 15947919

[11] Buch ER, Young S, Contreras-Vidal JL (2003) Visuomotor adaptation in normal aging. Learning & memory (Cold Spring Harbor, NY) 10: 55–63. 10.1101/lm.50303 PMC196655 12551964

[12] Baugh LA, Lawrence JM, Marotta JJ (2011) Novel claustrum activation observed during a visuomotor adaptation task using a viewing window paradigm. Behavioural brain research. 10.1016/j.bbr.2011.05.009 21621558

[13] Baugh LA, Marotta JJ (2009) When what’s left is right: visuomotor transformations in an aged population. PloS one 4: e5484. 19436727 10.1371/journal.pone.0005484 PMC2677156

[14] Baugh LA, Marotta JJ (2007) A new window into the interactions between perception and action. Journal of neuroscience methods 160: 128–134. 17156849 10.1016/j.jneumeth.2006.09.002

[15] Just MA, Carpenter PA, Woolley JD (1982) Paradigms and processes in reading comprehension. Journal of experimental psychologyGeneral 111: 228–238. 10.1037//0096-3445.111.2.228 6213735

[16] Osaka N, Oda N (1994) Moving window generator for reading experiments. Behav Res Meth Ins C 26: 49–53.

[17] van Diepen PM, Wampers M (1998) Scene exploration with Fourier-filtered peripheral information. Perception 27: 1141–1151. 10505194 10.1068/p271141

[18] Clower DM, Hoffman JM, Votaw JR, Faber TL, Woods RP, et al (1996) Role of posterior parietal cortex in the recalibration of visually guided reaching. Nature 383: 618–621. 8857536 10.1038/383618a0

[19] Rossetti Y, Rode G, Pisella L, Farne A, Li L, et al (1998) Prism adaptation to a rightward optical deviation rehabilitates left hemispatial neglect. Nature 395: 166–169. 9744273 10.1038/25988

[20] Marr D (1969) A theory of cerebellar cortex. The Journal of physiology 202: 437–470. 5784296 10.1113/jphysiol.1969.sp008820 PMC1351491

[21] Baizer JS, Kralj-Hans I, Glickstein M (1999) Cerebellar lesions and prism adaptation in macaque monkeys. Journal of neurophysiology 81: 1960–1965. 10200230 10.1152/jn.1999.81.4.1960

[22] Ito M (1993) Synaptic plasticity in the cerebellar cortex and its role in motor learning. The Canadian journal of neurological sciencesLe journal canadien des sciences neurologiques 20 Suppl 3 S70–74. 8334595

[23] Danckert J, Ferber S, Goodale MA (2008) Direct effects of prismatic lenses on visuomotor control: an event-related functional MRI study. The European journal of neuroscience 28: 1696–1704. 18973586 10.1111/j.1460-9568.2008.06460.x

[24] Luaute J, Schwartz S, Rossetti Y, Spiridon M, Rode G, et al (2009) Dynamic changes in brain activity during prism adaptation. The Journal of neuroscience : the official journal of the Society for Neuroscience 29: 169–178. 19129395 10.1523/JNEUROSCI.3054-08.2009 PMC6664918

[25] Diedrichsen J, Hashambhoy Y, Rane T, Shadmehr R (2005) Neural correlates of reach errors. The Journal of neuroscience : the official journal of the Society for Neuroscience 25: 9919–9931. 16251440 10.1523/JNEUROSCI.1874-05.2005 PMC1479774

[26] Chapman HL, Eramudugolla R, Gavrilescu M, Strudwick MW, Loftus A, et al (2010) Neural mechanisms underlying spatial realignment during adaptation to optical wedge prisms. Neuropsychologia 48: 2595–2601. 20457170 10.1016/j.neuropsychologia.2010.05.006

[27] Rabe K, Livne O, Gizewski ER, Aurich V, Beck A, et al (2009) Adaptation to visuomotor rotation and force field perturbation is correlated to different brain areas in patients with cerebellar degeneration. Journal of neurophysiology 101: 1961–1971. 19176608 10.1152/jn.91069.2008

[28] Kagerer FA, Contreras-Vidal JL, Bo J, Clark JE (2006) Abrupt, but not gradual visuomotor distortion facilitates adaptation in children with developmental coordination disorder. Human movement science 25: 622–633. 17011655 10.1016/j.humov.2006.06.003

[29] Newport R, Schenk T (2012) Prisms and neglect: what have we learned? Neuropsychologia 50: 1080–1091. 22306519 10.1016/j.neuropsychologia.2012.01.023

