# Supplementary material for: Behavioural Distinction between Strategic Control and Spatial Realignment during Visuomotor Adaptation in a Viewing Window Task
Source: PLoS One. 2012 Nov 16;7(11):e48759. doi: 10.1371/journal.pone.0048759 (PMC3500248; doi:10.1371/journal.pone.0048759)
Supplement: Supporting Information S1 — Supplementary methods and results. We examined behaviour of all of the 58 recruited participants in a separate analysis. The analysis methods and results are described. (DOCX) [file pone.0048759.s001.docx]

**S1. Supplementary Methods and Results**

In the current study, we considered only accurately identified trials as a method to address the influence of guessing. As a result, we set an accuracy threshold to include data from participants who were accurate at least 60% of the trials. The result was the exclusion of data from 18 participants. While this threshold is one method of assuring quality of the data, it is more dependent on the perceptual component of the Viewing Window task that the motor component which is of interest. While a threshold that is more specific to the motor component would be more desirable, this is also more subjective and so would be worthy of more study on its own to determine what an appropriate measure and threshold would be. To examine the influence of our accuracy threshold, we re-examined data from all 58 participants using the same statistical tests detailed in the methods section but including experimental trials regardless of accuracy. This resulted in asymmetric group sizes (Gradual Rotation, n=33; Sudden Rotation n=25). The Z scores for each trial off all participants were determined for each measure and outliers (Z>2.5 or Z<-2.5) were removed.

A one-way Anova of measures between groups revealed significantly faster velocities in the gradual rotation group(p=.000, F=36.138, df=1 ), no significant difference in scan time (p=2.61, F= 1.62, df 1), and that the sudden rotation group spent significantly more time off the object (p=0.23, F=5.163, df 1).

Examination of behaviour using a repeated measures general linear model over time [2x3, Group (Gradual vs Sudden) x Corrected Measure by Phase (Early distortions, Late distortions, Post distortions)] revealed that while there were significant changes in velocity over the normalized velocities over the phase of the experiment (p=0.004, F=6.896, df 1.546, alpha 0.05), there was no significant interaction with Rotation group (p=0.100, F=2.516, df 1.546, alpha 0.05). The interaction between phase and scan time approached significance (p=0.056). A significant interaction between phase and the time spent off the object was found (p=0.026; F=4.585; df 1.328, alpha 0.05).
